# Association between tongue, lips position and breathing in newborns

**DOI:** 10.1590/2317-1782/20232022049en

**Published:** 2023-10-13

**Authors:** Silvia Márcia Andrade Campanha, Roberta Lopes de Castro Martinelli, Durval Batista Palhares

**Affiliations:** 1 SerVoz- Saúde & Comunicação - Belo Horizonte (MG), Brasil.; 2 Hospital Santa Therezinha - Brotas (SP), Brasil.; 3 Programa de Pós-graduação em Saúde e Desenvolvimento na Região Centro-Oeste, Departamento de Pediatria, Faculdade de Medicina - FAMED, Universidade Federal de Mato Grosso do Sul - UFMS - Campo Grande (MS), Brasil.

**Keywords:** Breathing, Newborn, Tongue, Nasal obstruction, Mouth breathing

## Abstract

**Purpose:**

To verify the association between breathing nasal expiratory flow and posture of lips and tongue at rest, presence of repeated forward movements of the tongue and maternal complaint of respiratory difficulty in the newborn in the first days of life.

**Method:**

A observational study was carried out in 130 babies, in a university hospital. Included newborn with Apgar score greater than or equal to 8 in exclusive breast milk. It was the following data: position of lips and tongue at rest, nasal expiratory flow and maternal complaint of difficulty in breathing in the newborn. The data were subjected to statistical analysis using the tests, Fisher's exact test and the Chi-Square test, adopting a significance level of 5% .

**Results:**

there was a significant association between maternal complaint of newborn difficulty breathing with repeated forward tongue movements and nasal expiratory flow; tongue position with resting lips position at rest, repeated tongue forward movements with nasal expiratory flow and tongue position at rest; nasal expiratory flow exit with tongue position at rest.

**Conclusion:**

Symmetrical nasal expiratory flow is associated with an elevated tongue position and closed lips at rest; on the other hand, increased and/or absent nasal expiatory flow in one nostril is associated with maternal complaints of difficulty in breathing, open/ half-open lips position and low tongue position during rest, as well as, repeated forward tongue movements.

## INTRODUCTION

The newborn's breathing is predominantly nasal from birth to six months of life^(1)^, when the lips are kept closed and the tongue is elevated on the palate to prevent air from entering through the mouth^(2)^. This condition is possible due to the action of the lips and tongue muscles that reinforce the nasal breathing circuit to favor adequate coordination between sucking, swallowing and breathing during breastfeeding^(2,3)^.

In newborns, the upper airway is shorter and narrower when compared to adults, and any inflammation of the nasal mucosa, the most common cause of nasal obstruction in this population, can impair nasal breathing^(4,5)^.

Possible anatomofunctional alterations of the nose, as well as the physiological phenomenon of cyclical and rhythmic alternation of nasal congestion and decongestion of the nasal cavities, can interfere with the nasal expiratory flow, leading the newborn to present oral breathing for short periods, with consequent breathing effort and higher energy expenditure^(6)^.

Any breathing alteration, including nasal obstruction, can bring multiple consequences in the short and long term, affecting not only breathing, but also suction and craniofacial development^(4,7)^.

Nasal obstruction in newborns is a common complaint of mothers. However, the diagnosis often gets unnoticed by health professionals^(6)^. But, when the diagnosis of nasal obstruction is made, a detailed anamnesis and a clinical examination are used^(5)^.

The assessment of nasal patency is also recommended. In this case, the analysis of the nasal expiratory flow is used to identify a possible nasal obstruction and the predominant breathing mode^(8,9)^. Thus, the evaluation using the Altmann’s millimeter nasal mirror, adapted from the Glatzel mirror, is used by professionals in clinical practice to the analysis of the nasal expiratory flow, being a quick and non-invasive exam that requires minimal cooperation of the patient^(9-13)^.

Therefore, this study aimed to verify the association between nasal expiratory flow and posture of the lips and tongue at rest, the presence of repeated forward movements of the tongue and maternal complaints of breathing difficulty of the newborn in the first days of life.

## METHOD

This was an observational study, carried out with clinically stable newborns in the rooming-in of a University Hospital. This research was approved by the Ethics Committee under nº1. 514,715 of Federal University of Mato Grosso do Sul and all the responsible people for the newborns were informed about the procedures and signed the Informed Consent Form.

Initially, the sample calculation was performed considering a significance level of 5% and test power of 90%, being the average effect size based on the hypotheses of association between the study variables. The result showed the need to evaluate 117 newborns. Considering possible sample losses, a sample size of 130 babies was calculated, who were evaluated between 1 and 5 days old in the Rooming-in Room of a University Hospital.

The inclusion criteria in the study were: full-term newborns, with an APGAR score greater than or equal to eight, on exclusive breastfeeding. The study excluded indigenous and quilombo communities, newborns with perinatal complications, presence of craniofacial anomalies, neurological diseases, genetic syndromes visible at the time of evaluation and newborns receiving artificial feeding and the ones with unstable clinical conditions.

Assessments were performed between 24 hours after birth and 5 days of the newborn's life. Data collection was carried out by the researcher and by three properly trained speech-language pathologists from the institution's team. For this phase, a pilot study was carried out, with the participation of 14 newborns, whose evaluations were discussed by the speech-language pathologists, reaching a consensus on all the observed items.

Initially, an interview was conducted with the mother/guardian, asking whether or not the newborn had breathing difficulty. The answer options were yes or no for the following question: “Is the baby having difficulty in breathing through the nose?”.

Then, with the newborn sleeping in the crib, the position of the lips at rest was observed for 20 minutes, which could be closed or open/half-open. An evaluation of the tongue position at rest was also carried out, at 3 different moments, being at 5, 10 and 20 minutes, and it could be high or low in the oral cavity. Similar results were observed in the three moments. For this assessment, the examiner stood in front of the newborn, resting her gloved thumb on the chin region and lowering the mandible and lower lip, as proposed by Martinelli et al.^(14,15)^.

After that, the nasal expiratory flow was measured using the Nasal Mirror for Babies, designed to evaluate the newborns in this research. The Nasal Mirror for Babies was made with a mirrored polycarbonate sheet in a smaller size, with five centimeters in height and length and, internally, 25 squares of one centimeter^(16)^. These measurements were based on the newborns' orofacial measurements obtained in the pilot study^(16)^.

**Figure 1:**
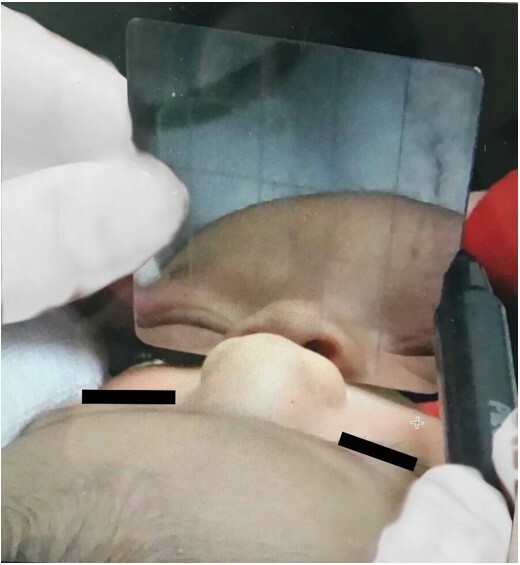
Measurement of nasal expiratory flow in newborn

**Figure 2:**
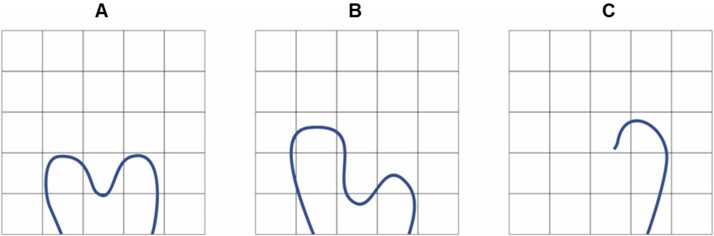
Measurement of nasal expiratory flow in newborn: A - Symmetric; B - Greater in one nostril; C - Absent in one nostril

To assess the expiratory flow, the nasal mirror was positioned at a 90º angle in relation to the nasal philtrum (Figure 1), with the baby resting on the mother's arm, close to the breastfeeding position, being considered symmetrical, greater in one nostril or absent, as shown in Figure 2. This assessment was performed before breastfeeding and with the newborn sleeping.

**Figure 3:**
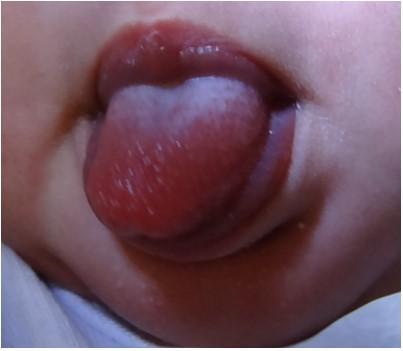
Forward movement of the tongue performed by the newborn

Then, with the newborn already awake, the presence or absence of repeated forward movements of the tongue was observed (Figure 3).

In this study, the measurement of nasal expiratory flow was considered as independent variables, and the dependent variables were: position of the lips and tongue at rest, presence or absence of repeated forward movements of the tongue and maternal complaint of respiratory difficulty in the newborn.

Data were entered into an Excel spreadsheet and submitted to the statistical analysis using the statistical program IBM SPSS Statistics (Statistical Package for the Social Sciences), version 25.0, using Fisher's Exact test and the Chi-Square test, considering the level significance of 5%.

## RESULTS

Of the 130 newborns evaluated, 70 (54%) were female and 60 (46%) were male, and 39 (30%) mothers reported complaints about the newborn's difficulty in breathing. As for the position of the lips and tongue at rest, 19 (15%) presented open or half-open lips and 34 (26%) kept their tongue low during rest in the oral cavity. Of the 130 newborns, 14 (11%) presented repeated forward tongue movements, 41 (31.5%) had greater nasal expiratory flow in one nostril and 18 (13.8%) had no flow in one nostril.

Table 1 shows that newborns whose mothers reported the difficulty in breathing had greater or absent expiratory flow in one nostril, and those whose mothers did not report difficulty in breathing had more symmetrical expiratory flow (p<0.001). When comparing the nasal expiratory flow of the newborns, the position of the tongue and lips at rest and the repeated forward movements of the tongue, a significant association was found between the nasal expiratory flow and the position of the tongue, being that newborns with symmetrical expiratory flow tended to position the tongue high, while newborns with absent nasal expiratory flow tended to position the tongue low at rest.

**Table 1:** Association between nasal expiratory flow, position of the tongue and lips at rest and repeated forward movements of the tongue

**Variables**		**Nasal Expiratory Flow**						**P-value**
		**Symmetric**		**Greater in one nostril**		**Absent in one nostril**		
Maternal complaint of difficulty for the newborn to breathe	No	62	87.3%	27	65.9%	2	11.1%	<0.001^(^1^)^
	Yes	9	12.7%	14	34.1%	18	88.9%	
Position of lips at rest	Closed	63	56.8%	34	30.6%	14	12.6%	0.342^(1)^
	Open/Half-open	8	42.1%	7	36.8%	4	21.1%	
Position of tongue at rest	High	58	60.4%	32	33.3%	6	6.3%	<0.001^(^2^)^
	Low	13	38.2%	9	26.5%	12	35.3%	
Repeated forward movements of the tongue	No	70	60.3%	35	30.2%	11	9.5%	<0.001^(1)^
	Yes	1	7.1%	6	42.9%	7	50.0%	

1 Fisher's Exact Test;

2 Chi-Square Test

An association was also found between the nasal expiratory flow (<0.001) and repeated forward movements of the tongue (<0.001), showing that newborns who had absent nasal expiratory flow in one nostril presented repeated forward movements of the tongue.

## DISCUSSION

This research was conducted with the aim of verifying the association between breathing, considering the nasal expiratory flow, and tongue and lips position at rest in clinically stable newborns in the first days of life.

A significant association was found between the mother's complaint of the newborn difficulty in breathing and the nasal expiratory flow. Greater or absent nasal expiratory flow in one nostril was more frequent in newborns whose mothers reported difficulty in breathing of the newborn. Anatomophysiological alterations of the nose, as a physiological phenomenon of cyclical and rhythmic alternation of congestion and decongestion of the nasal cavities, due to the immaturity of the vasomotor reaction of the nasal mucosa in newborns, and a nasal obstruction can lead to long periods of nasal obstruction - with maternal complaints^(6)^.

The findings of this research also showed that most of the newborns who presented a low tongue position kept their lips open/half-open at rest, with absent expiratory flow in one nostril. On the other hand, newborns who presented an elevated tongue position kept their lips closed at rest and symmetrical nasal expiratory flow. Both the half-open lips position and the low tongue in the oral cavity during rest are associated with oral breathing^(17)^, which can be caused by nasal obstruction^(18)^.

There was a significant association between repeated tongue forward movements and greater or absent nasal expiratory flow on one side, low tongue position and the mother's complaint of difficulty for the newborn to breathe. No studies were found that had associated the aspects mentioned above to compare the findings. However, there is a description in the literature about the influence of the tongue muscles on breathing^(19-21)^. One study showed an increase in the genioglossus muscle activation through electromyography recording in the face of an airflow limitation in patients with obstructive sleep apnea. Another study carried out with animals showed an increase in the activity of the extrinsic and intrinsic muscles of the tongue in response to the airway obstruction^(22)^. The coactivation of these muscles occurs due to the disposition of the intrinsic muscle fibers in relation to the extrinsic muscles of the tongue. This mechanism has the potential to increase tongue stiffness and contribute to the reopening of the airways by pushing the tongue forward^(22)^.

The genioglossus muscle, which is part of the extrinsic musculature of the tongue, is considered the respiratory muscle of the tongue, being responsible for moving the hyoid bone and the base of the tongue forward during inspiration to expand the oropharyngeal region^(20-23)^. Some authors also reported that the activation of the genioglossus muscle may be related to the airflow resistance, that is, in oral breathing, mouth opening associated with the lowering of the mandible favors lower oropharyngeal resistance, with increased activity of the genioglossus muscle^(24)^.

Thus, understanding the role of the tongue in breathing is considerably important as it plays an important role in regulating the resistance of the upper airways^(25)^, and alteration in tongue movement is an aggravating factor for obstructive sleep apnea^(26)^.

Therefore, based on the literature and the results obtained in this study, we can infer that, in the presence of a nasal obstruction, that is, an alteration of the nasal expiratory flow, the newborn can remain with the tongue low, presenting repeated forward movements of the tongue to open the airways. These movements will help to open the lips, favoring the entry of air through the mouth.

In view of these findings, the observation of the position of the lips and tongue at rest, the nasal expiratory flow and the presence of repeated forward movements of the tongue in newborns are variables to be included in evaluation protocols for clinically stable newborns, contributing to the prevention of mouth breathing.

The reduced number of newborns with absent expiratory flow or flow in only one nostril was a limitation of this study. Other studies are needed to assess the factors that may interfere with nasal permeability in clinically stable newborns.

## CONCLUSION

In the first days of life, the symmetrical nasal expiratory flow in newborns is associated with the elevated position of the tongue and closed lips at rest; on the other hand, greater and/or absent nasal expiratory flow in one nostril is associated with maternal complaint of difficulty for the newborn to breathe, open/half-open lips position and low tongue position at rest, and presence of repeated forward movements of the tongue.
