# Supplementary material for: Association between tongue, lips position and breathing in newborns
Source: Codas. 2023 Oct 13;35(5):e20220049. doi: 10.1590/2317-1782/20232022049en (PMC10688296; doi:10.1590/2317-1782/20232022049en)
Supplement: Translation

# Translation: Associação entre posição de língua, lábios e respiração em recém-nascidos

**DOI:** 10.1590/2317-1782/20232022049pt

**Authors:** Silvia Márcia Andrade Campanha, Roberta Lopes de Castro Martinelli, Durval Batista Palhares

**Affiliations:** 1 SerVoz- Saúde & Comunicação - Belo Horizonte (MG), Brasil.; 2 Hospital Santa Therezinha - Brotas (SP), Brasil.; 3 Programa de Pós-graduação em Saúde e Desenvolvimento na Região Centro-Oeste, Departamento de Pediatria, Faculdade de Medicina - FAMED, Universidade Federal de Mato Grosso do Sul - UFMS - Campo Grande (MS), Brasil.

**Keywords:** Respiração, Recém-nascido, Língua, Obstrução Nasal, Respiração Bucal

## Abstract

**Objetivo:**

verificar a associação entre fluxo expiratório nasal e postura de lábios e língua no repouso, presença de movimentos repetidos de anteriorização de língua e queixa materna de dificuldade respiratória do recém-nascido nos primeiros dias de vida.

**Método:**

estudo observacional, realizado com 130 recém-nascidos do alojamento conjunto de um hospital Universitário. Foram incluídos recém-nascidos a termo, com idade entre 1 e 5 dias de vida, APGAR maior ou igual a oito, em aleitamento materno exclusivo. Foram coletados os seguintes dados: posição de lábios e língua no repouso, fluxo expiratório nasal, presença de movimentos repetidos de anteriorização de língua e queixa materna de dificuldade do recém-nascido para respirar. Os dados foram submetidos à análise estatística, sendo aplicado o Teste Exato de Fisher e o Qui-Quadrado, adotando-se o nível de significância de 5%.

**Resultados:**

Há uma associação significativa entre queixas maternas de dificuldade do recém-nascido para respirar pelo nariz com movimentos repetidos de anteriorização de língua e fluxo expiratório nasal; posição de língua com posição de lábios no repouso, movimentos repetidos de anteriorização de língua com fluxo expiratório nasal e posição de língua no repouso; fluxo expiratório nasal com posição de língua no repouso.

**Conclusão:**

O fluxo expiratório nasal simétrico está associado com a posição de língua elevada e lábios fechados no REPOUSO; por outro lado, o fluxo expiratório nasal maior e/ou ausente em uma narina está associado com queixa materna de dificuldade do recém-nascido para respirar, posição de lábios abertos/entreabertos E posição de língua baixa no repouso, bem como, com movimentos repetidos de anteriorização de língua.

## INTRODUÇÃO

A respiração do recém-nascido é predominantemente nasal desde o nascimento até seis meses de vida^(1)^, quando mantém os lábios fechados e a língua elevada no palato para evitar a entrada de ar pela boca^(2)^. Essa condição é possível em decorrência da ação dos músculos dos lábios e da língua que reforçam o circuito da respiração nasal para favorecer uma adequada coordenação entre sucção, deglutição e respiração durante a amamentação^(2,3)^.

Em recém-nascidos, a via aérea superior é mais curta e estreita quando comparada a dos adultos, sendo que qualquer inflamação da mucosa nasal, causa mais comum de obstrução nasal nesta população, pode comprometer a respiração nasal^(4,5)^.

Possíveis alterações anatomofuncionais do nariz, bem como, o fenômeno fisiológico de alternância cíclica e rítmica de congestão e descongestão nasal das fossas nasais podem interferir no fluxo expiratório nasal, levando o recém-nascido a apresentar uma respiração oral por curtos períodos, com consequente esforço respiratório e maior gasto energético^(6)^ **_._**

Qualquer alteração respiratória, incluindo a obstrução nasal, pode acarretar múltiplas consequências a curto e longo prazo, afetando não apenas a respiração, mas também a sucção e o desenvolvimento craniofacial^(4,7)^.

A obstrução nasal em recém-nascidos é uma queixa comum das mães, porém, muitas vezes, seu diagnóstico passa despercebido pelos profissionais de saúde^(6)^_._ Entretanto, quando o diagnóstico da obstrução nasal é realizado, utiliza-se uma anamnese detalhada, bem como, um exame clínico minucioso^(5)^.

A avaliação da patência nasal também é recomendada, sendo utilizada, nesse caso, a análise do fluxo expiratório nasal para identificar uma possível obstrução nasal e o modo respiratório predominante^(8,9)^. Assim, a avaliação utilizando o espelho nasal Milimetrado de Altmann, adaptado do espelho de Glatzel é utilizado por profissionais na prática clínica para análise do fluxo expiratório nasal, sendo um exame rápido e não invasivo, além de necessitar de mínima cooperação do paciente^(9-13)^.

Sendo assim, este estudo teve como objetivo verificar a associação entre fluxo expiratório nasal e postura de lábios e língua no repouso, presença de movimentos repetidos de anteriorização de língua e queixa materna de dificuldade respiratória do recém-nascido nos primeiros dias de vida.

## MÉTODO

Este foi um estudo observacional, realizado com recém-nascidos clinicamente estáveis do Alojamento Conjunto de um Hospital Universitário. Esta pesquisa foi aprovada pelo Comitê de Ética sob n^o^1. 514.715 da Universidade Federal de Mato Grosso do Sul, sendo que todos os responsáveis foram informados sobre os procedimentos e assinaram o Termo de Consentimento Livre e Esclarecido.

Inicialmente, foi realizado o cálculo amostral considerando-se o nível de significância de 5% e poder de teste de 90%, sendo o tamanho do efeito médio baseado nas hipóteses de associação entre as variáveis do estudo. O resultado mostrou a necessidade da avaliação de 117 recém-nascidos. Considerando as possíveis perdas da amostra, chegou-se ao cálculo amostral de 130 bebês, que foram avaliados com idade entre 1 e 5 dias de vida no Alojamento Conjunto de um Hospital Universitário.

Os critérios de inclusão no estudo foram: recém-nascidos a termo, com escore de APGAR maior ou igual a oito, em aleitamento materno exclusivo. Foram excluídos da pesquisa, população indígena e quilombola, recém-nascidos com complicações perinatais, presença de anomalias craniofaciais, doenças neurológicas, síndromes genéticas visíveis no momento da avaliação e alimentação artificial e recém-nascidos com condições clínicas instáveis.

As avaliações foram realizadas entre 24 horas após o nascimento e 5 dias de vida do recém-nascido. A coleta de dados foi realizada pela pesquisadora e por três fonoaudiólogas da equipe da Instituição, devidamente treinadas. Para essa fase, foi realizado um estudo piloto, com a participação de 14 recém-nascidos, cujas avaliações foram discutidas pelas fonoaudiólogas chegando ao consenso de todos os itens observados.

Inicialmente, foi realizada uma entrevista com a mãe/responsável, sendo questionada a presença ou não de dificuldade do recém-nascido para respirar. As opções de resposta foram sim ou não para o seguinte questionamento: “o bebê está com dificuldade para respirar pelo nariz?”.

Em seguida, com o recém-nascido dormindo no berço, foi observada a posição de lábios no repouso, durante 20 minutos, podendo estar fechados ou abertos/entreabertos. Também foi realizada a avaliação da posição da língua em repouso, em 3 momentos distintos, ou seja, aos 5, 10 e 20 minutos, podendo estar elevada ou baixa na cavidade oral. Foram observados resultados semelhantes nos três momentos. Para essa avaliação, a examinadora se posicionava à frente do recém-nascido, apoiando o dedo polegar enluvado na região do mento e realizando o abaixamento da mandíbula e do lábio inferior, conforme proposto por Martinelli e colaboradores^(14,15)^.

Em seguida, foi realizada a medida do fluxo expiratório nasal por meio do Espelho Nasal para Bebês, idealizado para avaliar os recém-nascidos desta pesquisa. O Espelho Nasal para Bebês foi confeccionado com chapa de policarbonato espelhada nasal em tamanho menor, com cinco centímetros de altura e comprimento, e internamente 25 quadrados de um centímetro^(16)^. Estas medidas foram baseadas nas medidas orofaciais dos recém-nascidos obtidas no estudo piloto^(16)^.

Para a avaliação do fluxo expiratório, o espelho nasal foi posicionado em um ângulo de 90^o^ em relação ao filtro nasal (Figura 1), com o bebê apoiado no braço da mãe, próximo à posição de amamentar, sendo considerado simétrico, maior em uma narina ou ausente, conforme a Figura 2. Essa avaliação foi realizada antes da amamentação e com o recém-nascido dormindo.

**Figura 1:** Medida do Fluxo Expiratório Nasal no recém-nascido

**Figura 2:** Medida do Fluxo Expiratório Nasal no recém-nascido: A - Simétrico; B - Maior em uma narina; C- Ausente em uma narina

Em seguida, com o recém-nascido já em estado de alerta, foi observada a presença ou ausência de movimentos repetidos de anteriorização de língua (Figura 3).

**Figura 3:** Movimento de anteriorização de língua realizado pelo recém-nascido

Neste estudo considerou-se como variáveis independentes a medida do fluxo expiratório nasal e as variáveis dependentes foram: posição de lábios e língua em repouso, presença ou ausência de movimentos repetidos de anteriorização de língua, queixa materna de dificuldade respiratória pelo recém-nascido.

Os dados foram inseridos em uma planilha Excel e submetidos à análise estatística por meio do programa estatístico IBM SPSS Statistics (Statistical Package for the Social Sciences), versão 25.0, sendo utilizado o teste Exato de Fisher e o teste Qui-Quadrado, considerando o nível de significância de 5%.

## RESULTADOS

Dos 130 recém-nascidos avaliados, 70 (54%) eram do sexo feminino e 60 (46%) do sexo masculino, sendo que 39 (30%) mães relataram queixa quanto à dificuldade do recém-nascido para respirar. Quanto à posição de lábios e língua no repouso, 19 (15%) apresentaram os lábios abertos ou entreabertos e 34 (26%) mantiveram a língua baixa na cavidade oral. Dos 130 recém-nascidos, 14 (11%) apresentaram movimentos repetidos de anteriorização de língua, 41 (31,5%) fluxo expiratório nasal maior em uma narina e 18 (13,8%) fluxo ausente em uma narina.

A Tabela 1 mostra que recém-nascidos cujas mães relataram sua dificuldade para respirar apresentaram o fluxo expiratório maior ou ausente em uma narina, e aqueles cujas mães não relataram sua dificuldade para respirar apresentaram o fluxo expiratório mais simétrico (p<0,001). Quando comparados o fluxo expiratório nasal dos recém-nascidos, a posição de língua e lábios no repouso e os movimentos repetidos de anteriorização de língua, encontrou-se associação significativa entre o fluxo expiratório nasal e a posição de língua no repouso, sendo que recém-nascidos com o fluxo expiratório simétrico tenderam a posicionar a língua elevada, enquanto Recém-nascidos com fluxo expiratório nasal ausente tenderam a posicionar a língua baixa no repouso.

**Tabela 1:** Associação entre fluxo expiratório nasal, posição de língua e lábios no repouso e movimentos repetidos de anteriorização de língua

**Variáveis**		**Fluxo Expiratório Nasal**						**Valor-p**
		**Simétrico**		**Maior em uma narina**		**Ausente em uma narina**		
Queixa materna de dificuldade do recém-nascido para respirar	Não	62	87,3%	27	65,9%	2	11,1%	<0,001^(1)^
	Sim	9	12,7%	14	34,1%	18	88,9%	
Posição de lábios no repouso	Fechados	63	56,8%	34	30,6%	14	12,6%	0,342^(1)^
	Abertos/Entreabertos	8	42,1%	7	36,8%	4	21,1%	
Posição de língua no repouso	Elevada	58	60,4%	32	33,3%	6	6,3%	<0,001^(2)^
	Baixa	13	38,2%	9	26,5%	12	35,3%	
Mov. repetidos de anteriorização de língua	Não	70	60,3%	35	30,2%	11	9,5%	<0,001^(1)^
	Sim	1	7,1%	6	42,9%	7	50,0%	

1 Teste Exato de Fisher;

2 Teste Qui-Quadrado

Foi encontrado também uma associação entre o fluxo expiratório nasal (<0,001) e movimentos repetidos de anteriorização de língua (<0,001), evidenciando que recém-nascidos que apresentaram fluxo expiratório nasal ausente em uma narina apresentaram movimentos repetidos de anteriorização da língua.

## DISCUSSÃO

Esta pesquisa foi conduzida com o objetivo de verificar a associação entre respiração, considerando o fluxo expiratório nasal, e posição de língua e lábios no repouso em recém-nascidos clinicamente estáveis nos primeiros dias de vida.

Foi encontrada associação significativa entre a queixa da mãe de dificuldade do recém-nascido para respirar com o fluxo expiratório nasal. O fluxo expiratório nasal maior ou ausente em uma narina foi mais frequente nos recém-nascidos cujas mães relataram queixa de dificuldade do recém-nascido para respirar. Alterações anatomofisiológicas do nariz, como um fenômeno fisiológico de alternância cíclica e rítmica da congestão e descongestão das fossas nasais, devido à imaturidade da reação vasomotora da mucosa nasal em recém-nascidos e uma obstrução nasal podem levar a períodos longos de obstrução nasal^-^ com queixas maternas^(6)^.

Os achados dessa pesquisa também mostraram que nos recém-nascidos que apresentaram posição de língua baixa, a maioria manteve os lábios abertos/entreabertos no repouso, com o fluxo expiratório ausente em uma narina. Por outro lado, recém-nascidos que apresentaram posição da língua elevada, mantiveram os lábios fechados no repouso e fluxo expiratório nasal simétrico. Tanto a posição de lábios entreabertos, quanto a língua baixa na cavidade oral durante o repouso estão associadas com a respiração oral^(17)^, que pode ser causada por uma obstrução nasal^(18)^_._

Houve associação significativa entre movimentos repetidos de anteriorização de língua com o fluxo expiratório nasal maior ou ausente de um lado, com a posição de língua baixa no repouso e com a queixa materna de dificuldade do recém-nascido para respirar. Não foram encontrados estudos que tenham associado os aspectos mencionados acima para comparação dos achados. Entretanto, há descrição na literatura sobre a influência dos músculos da língua na respiração^(19-21)^. Um estudo mostrou um aumento na ativação do músculo genioglosso por meio do registro da eletromiografia diante de uma limitação do fluxo aéreo em pacientes com apnéia obstrutiva do sono. Outro estudo realizado com animais mostrou um aumento da atividade da musculatura extrínseca e intrínseca da língua em resposta à obstrução das vias aéreas^(22)^. A coativação destes músculos acontecem devido à disposição das fibras musculares intrínsecas em relação a musculatura extrínseca da língua. Este mecanismo tem o potencial para aumentar a rigidez lingual e contribuir para a reabertura das vias respiratórias por meio da anteriorização da língua^(22)^.

O músculo genioglosso, que faz parte da musculatura extrínseca da língua, é considerado o músculo respiratório da língua, sendo responsável por mover o osso hioide e a base da língua anteriormente durante a inspiração para ampliar a região orofaríngea^(20-23)^. Alguns autores relataram também que a ativação do músculo genioglosso pode estar relacionada com a resistência do fluxo aéreo, ou seja, em uma respiração oral, a abertura de boca associada à descida da mandíbula, favorece uma menor resistência orofaríngea, com aumento da atividade do músculo genioglosso^(24)^.

Assim, a compreensão da função da língua na respiração é de considerável importância, pois desempenha um papel relevante na regulação da resistência das vias aéreas superiores^(25)^, e alteração na movimentação da língua é um agravante para a apneia obstrutiva do sono^(26)^.

Portanto, com base na literatura e nos resultados obtidos nesse estudo podemos inferir que, na presença de uma obstrução nasal, ou seja, de uma alteração do fluxo expiratório nasal, o recém-nascido pode permanecer com a língua baixa, apresentando movimentos repetidos de anteriorização de língua para abrir as vias respiratórias. Estes movimentos contribuirão para a abertura de lábios, favorecendo a entrada de ar pela boca.

Diante desses achados, a observação da posição de lábios e língua no repouso, o fluxo expiratório nasal, bem como a presença de movimentos repetidos de anteriorização de língua em recém-nascidos são variáveis a serem inseridas em protocolos de avaliação de recém-nascidos clinicamente estáveis, contribuindo para a prevenção da instalação da respiração oral.

O número reduzido de recém-nascidos com o fluxo expiratório ausente ou somente em uma narina foi uma limitação deste estudo. Outros estudos são necessários visando avaliar os fatores que podem interferir na permeabilidade nasal em recém-nascidos clinicamente estáveis.

## CONCLUSÃO

Nos primeiros dias de vida, o fluxo expiratório nasal simétrico em recém-nascidos está associado com a posição de língua elevada e lábios fechados no repouso; por outro lado, o fluxo expiratório nasal maior e/ou ausente em uma narina está associado com queixa materna de dificuldade do recém-nascido para respirar, posição de lábios abertos/entreabertos e posição de língua baixa no repouso, bem como, com presença de movimentos repetidos de anteriorização de língua.
